# A randomized prospective trial of the postoperative quality of life between laparoscopic uterine artery ligation and laparoscopy-assisted vaginal hysterectomy for the treatment of symptomatic uterine fibroids: clinical trial design

**DOI:** 10.1186/1745-6215-10-8

**Published:** 2009-01-29

**Authors:** Hee Seung Kim, Jae Weon Kim, Mi-Kyung Kim, Hyun Hoon Chung, Taek Sang Lee, Yong-Tark Jeon, Yong Beom Kim, Hye Won Jeon, Young Ho Yun, Noh Hyun Park, Yong Sang Song, Soon-Beom Kang

**Affiliations:** 1 Department of Obstetrics and Gynecology, Seoul National University College of Medicine, Seoul National University Hospital, Seoul, 110-744, Republic of Korea; 2 Department of Obstetrics and Gynecology, Seoul National University College of Medicine, Seoul National University Borame Hospital, Seoul, 156-707, Republic of Korea; 3 Department of Obstetrics and Gynecology, Seoul National University College of Medicine, Seoul National University Bundang Hospital, Seongnam, 463-707, Republic of Korea; 4 Quality of Cancer Care Branch, Research Institute for National Cancer Control and Evaluation, National Cancer Center, Goyang, 411-351, Republic of Korea

## Abstract

**Background:**

Laparoscopy-assisted vaginal hysterectomy is one of the definite methods for the treatment of symptomatic uterine fibroids with lesser intraoperative bleeding and shorter hospitalization compared with abdominal hysterectomy. However, laparoscopy-assisted vaginal hysterectomy cannot preserve uterus and can show postoperative complications by the change of pelvic structure. Thus, laparoscopic uterine artery ligation has been introduced for relieving the symptoms caused by uterine fibroids in place of hysterectomy. The current study was designed to compare postoperative quality of life between laparoscopic uterine artery ligation and laparoscopy-assisted vaginal hysterectomy, and to evaluate the efficacy of laparoscopic uterine artery ligation which can treat symptomatic uterine fibroids with the preservation of uterus.

**Methods and design:**

Patients enrolled the current study are randomized to laparoscopic uterine artery ligation or laparoscopy-assisted vaginal hysterectomy. The primary outcome is to compare postoperative quality of life between laparoscopic uterine artery ligation and laparoscopy-assisted vaginal hysterectomy using the European Organization for Research and Treatment of Cancer Quality of Life Questionnaire for Cancer patients version 3.0. Secondary outcomes are to evaluate the volume reduction of uterus, uterine fibroids and ovaries by the 2 treatments, to compare the improvement of subjective symptoms using 11-point symptom score and postoperative clinical outcomes between laparoscopic uterine artery ligation and laparoscopy-assisted vaginal hysterectomy, and to investigate the improvement of postoperative vaginal bleeding by laparoscopic uterine artery ligation.

**Discussion:**

Among treatment methods for symptomatic uterine fibroids with the preservation of uterus, laparoscopic uterine artery ligation is expected to have the efficacy like uterine artery embolization, which appeared to be safe for routine use with symptomatic relief. The current study fully recruited in June 2008 and the results will be available in June 2009. If there is no difference of postoperative QOL between laparoscopic uterine artery ligation and laparoscopy-assisted vaginal hysterectomy for the treatment of symptomatic uterine fibroids, the comparison of quality of life between laparoscopic uterine artery ligation and uterine artery embolization will be also needed as a surgical treatment for preserving uterus.

**Trial registration:**

Current Controlled Trials ISRCTN76790866

## Background

Uterine fibroids are the most common benign uterine tumors. They may be more common in women who are obese, and there appears to be an increased familial incidence [1]. Although fewer than one half of them are estimated to produce symptoms [2], uterine fibroids may cause different symptoms including menorrhagia, intermittent menstrual bleeding, pelvic pain, urinary frequency and constipation.

The management of uterine fibroids is dependent on the patient's age and proximity to anticipated menopause, symptoms, patient preference, and the experience and skills of the clinician. Although non-surgical treatment using gonadotropin-releasing hormone (GnRH) agonist is effective for reducing the size and symptoms of uterine fibroids, surgical treatment is also needed for patients with potential indications such as abnormal uterine bleeding unresponsive to hormone, urinary symptoms or signs such as hydronephrosis and infertility with uterine fibroids as the only abnormal finding.

Among surgical treatments, hysterectomy is a definite method for the treatment of symptomatic uterine fibroids. Especially, laparoscopy-assisted vaginal hysterectomy (LAVH) has more advantages including lesser intraoperative bleeding and shorter hospitalization than abdominal hysterectomy [3]. However, LAVH cannot preserve uterus and can show postoperative complications by the change of pelvic structure [4].

Thus, laparoscopic uterine artery ligation (LUAL) has been introduced in 2001 as an alternative technique for treating uterine fibroids, and significant reduction in the dominant fibroid size (average, 76%) and the uterine volume (average, 46%) were sonographically demonstrated [5]. Thereafter, some studies have reported that LUAL may be effective in relieving the symptoms caused by uterine fibroids, and this procedure can be used in place of hysterectomy [6,7]. Although of these results, there is no study for the comparison of postoperative quality of life (QOL) between LUAL and LAVH.

The current study has been designed to compare the postoperative QOL between LUAL and LAVH, and to evaluate the efficacy of LUAL which can treat symptomatic uterine fibroids with the preservation of uterus.

## Methods and design

### Study design

This is a multi-center randomized controlled trial comparing postoperative QOL between LUAL and LAVH for the treatment of symptomatic uterine fibroids. Eligible patients were randomized in a 1:2 ratio to undergo either LAUL or LAVH.

### Primary outcome

Postoperative QOL 12 months after surgical treatment are evaluated using the European Organization for Research and Treatment of Cancer Quality of Life Questionnaire for Cancer patients (EORTC QLQ-C30) version 3.0., which has been validated to the simplified Korean version [8]. After the evaluation of postoperative QOL, the results will be compared between LUAL and LAVH.

### Secondary outcomes

#### Volume reduction of uterus, uterine fibroids and ovaries by USG

The volume reduction of uterus, uterine fibroids and both ovaries is evaluated in patients treated with LUAL, whereas the volume reduction of only both ovaries is examined in those treated with LAVH.

Improvement of subjective symptoms11-point symptom score, ranging from -5 (markedly worse) to +5 (markedly better), is a method for evaluating either improvement or aggravation of symptoms which patients have, for example, menorrhagia, pain, gastrointestinal discomfort, etc [9]. It is applied in all patients for evaluating the improvement of subjective symptoms by surgical treatment.

#### Evaluation of postoperative vaginal bleeding

In patients treated with LUAL, postoperative vaginal bleeding is measured using a simple visual assessment technique [10]. It is measured monthly by use of the recording sheet including simple visual assessment technique by herself.

#### Postoperative clinical outcomes

Compared with preoperative status, postoperative clinical outcomes are compared between LUAL and LAVH as follows: anemia, hormonal status and serum CA-125 levels; operation time, hospitalization and recovery time to routine life; postoperative pain; conversion to laparotomy; satisfaction of postoperative sexual intercourse; complications associated with surgery.

### Eligibility

#### Inclusion criteria

• Age ≥ 40 years

• Premenopausal patients

• Patients who do not want conception any more

• Patients who agree to the current study protocol with informed consent

• Patients with more than 2 cm sized uterine fibroids on ultrasonography (USG)

• Patients with symptomatic uterine fibroids such as menorrhagia, dysmenorrhea, lower abdominal discomfort or pain, lower back pain and urological symptoms including dysuria and frequency

• Patients without underlying disease affecting QOL

• At least six months interval after last medication if patients have been treated with GnRH agonists.

#### Exclusion criteria

• Age < 40 years

• Patients with subserosal pedunculated uterine fibroids

• Pregnant patients

• Patients with pelvic inflammatory disease (PID) developed within one month

• Patients with suspicious adenomyosis by USG

• Patients contraindicated by surgical treatment

• Patients with previous history of myomectomy, hysterectomy, myolysis, uterine artery embolization

• Patients with underlying disease affecting QOL

• Less than six month's interval after last medication, if patients have been treated with GnRH agonist

### Interventions

#### Laparoscopic uterine artery ligation

After peril-umbilical tracer puncture and the infusion of CO_2 _gas, 2 or 3 tracer punctures are made additionally. Entering the peritoneal cavity, bilateral mesosalpinges including ovarian vessels are coagulated and ligated using hemoclips. Thereafter, right broad ligament is incised between right round ligament and fallopian tube, and the retroperitoneal space is exfoliated using non-traumatic forceps. After right internal iliac artery is seen, the branch of the artery, right uterine artery, is identified. Bipolar coagulation followed by the ligation using hemoclips is applied to right uterine artery. Left uterine artery is ligated according to the procedure for right uterine artery ligation.

#### Laparoscopy-assisted vaginal hysterectomy

After peri-umbilical trocar puncture and the infusion of CO_2 _gas, 2 or 3 trocar punctures are made additionally. Entering the peritoneal cavity, bilateral round ligaments, ovarian ligaments and fallopian tubes are ligated. The uterus is pulled down into the operative field by traction on the each lateral side of the cervical lip. The initial transverse incision through the anterior vaginal wall is made at the cervicovaginal junction and extended laterally on both sides to form encircling incision. The bladder is dissected off the cervix and lower uterine segment anteriorly, and bilateral bladder pillars are ligated. The exfoliation of bladder is advanced to the point where the vesicouterine (U-V) fold is seen, and the U-V fold is incised transversely and the peritoneal cavity is opened. The posterior vaginal wall is dissected off the cervix to the peritoneal reflection of the cul-de-sac. Bilateral cardinal ligaments, uterine vessels and uterosacral ligaments are ligated, and thereby the uterus is delivered through the anterior fornix using tenaculum.

## Outcome measurements

### Primary outcome

After patients are enrolled and randomized, we interview them using EORTC QLQ-C30 before surgical intervention for the evaluation of preoperative QOL. Since the validity of the EORTC QLQ-C30 has already been demonstrated in Republic of Korea [8], and the authors responsible for the validation recommended its usefulness in the current study, we decided to apply it for the evaluation of postoperative QOL in patients with symptomatic uterine fibroids.

**Figure 1:**
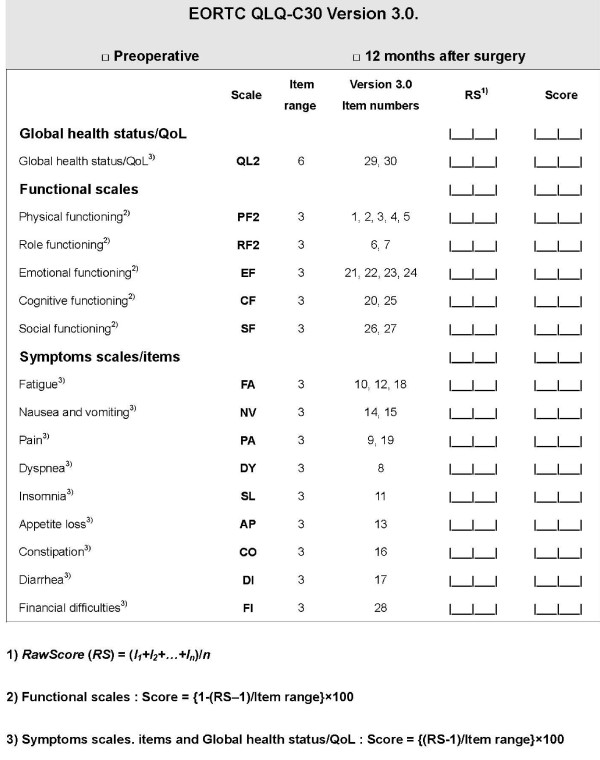
**European Organization for Research and Treatment of Cancer Quality of Life Questionnaire for Cancer patients (EORTC QLQ-C30) version 3.0. for comparing postoperative quality of life between laparoscopic uterine artery ligation and laparoscopy-assisted vaginal hysterectomy**.

The EORTC QLQ-C30 is a 30-item core-cancer-specific questionnaire-integrating system for assessing the health-related QOL in patients participating in international clinical trials [11]. The questionnaire incorporates five functional scales (physical, role, cognitive, emotional, and social), three symptom scales (fatigue, pain, and nausea and vomiting), a global health and QOL scale, and single items for the assessment of additional symptoms commonly reported by cancer patients (e.g., dyspnea, appetite loss, sleep disturbance, constipation, and diarrhea), as well as the perceived financial impact of the disease and treatment [12]. All items are scored on 4-point Likert scales, ranging from 1 ('not at all') to 4 ('very much'), with the exception of two items in the global health/QOL scale which use modified 7-point linear analog scales [11]. After 12 months, all enrolled patients will be interviewed, and the results of EORTC QLQ-C30 will be compared with preoperative results of it (Fig. 1).

### Secondary outcomes

#### Volume reduction of uterus, uterine fibroids and bilateral ovaries by USG

**Figure 2:**
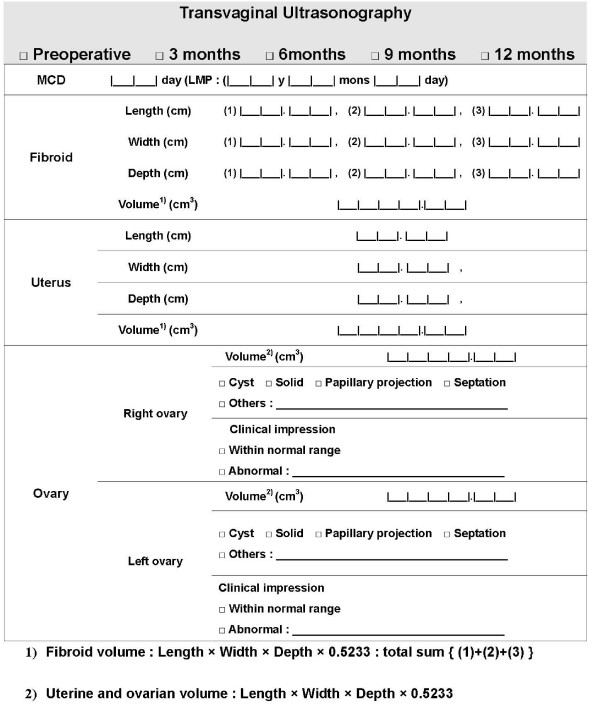
**Calculation of volumes of uterus, uterine fibroids and bilateral ovaries by use of transvaginal ultrasonography**.

The method for evaluating volumes of uterus, uterine fibroids and ovaries is as follows: Volume (cm^3^) = maximal sagittal diameter (length, cm) × maximal coronal diameter (width, cm) × maximal antero-posterior diameter (depth, cm) × 0.5233 [13]. Volumes of uterus and uterine fibroids are evaluated in patients treated with LUAL preoperatively and every 3 months, whereas volumes of bilateral ovaries are examined in all patients preoperatively and every 6 months (Fig. 2).

#### Improvement of subjective symptoms

**Figure 3:**
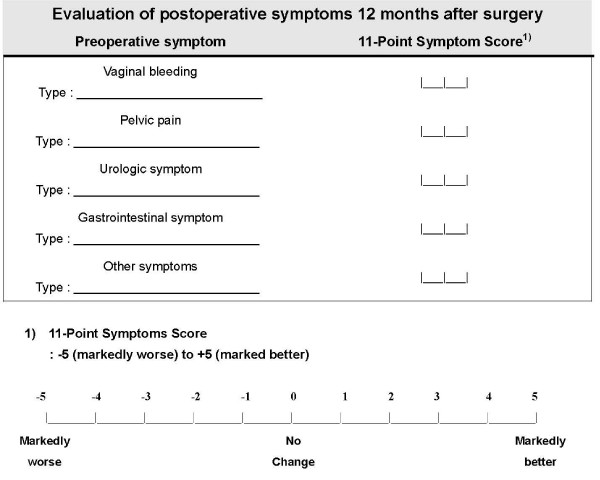
**11-point symptoms score for evaluating of postoperative symptoms 12 months after surgery**.

11-point symptom score are evaluated in all patients 12 months after surgery. Symptoms by uterine fibroids are divided as follows; vaginal bleeding: pelvic pain: urologic symptom; gastrointestinal symptom; other symptoms (Fig. 3). After the completion of the current study, the results are compared between LUAL and LAVH.

#### Evaluation of postoperative vaginal bleeding

**Figure 4:**
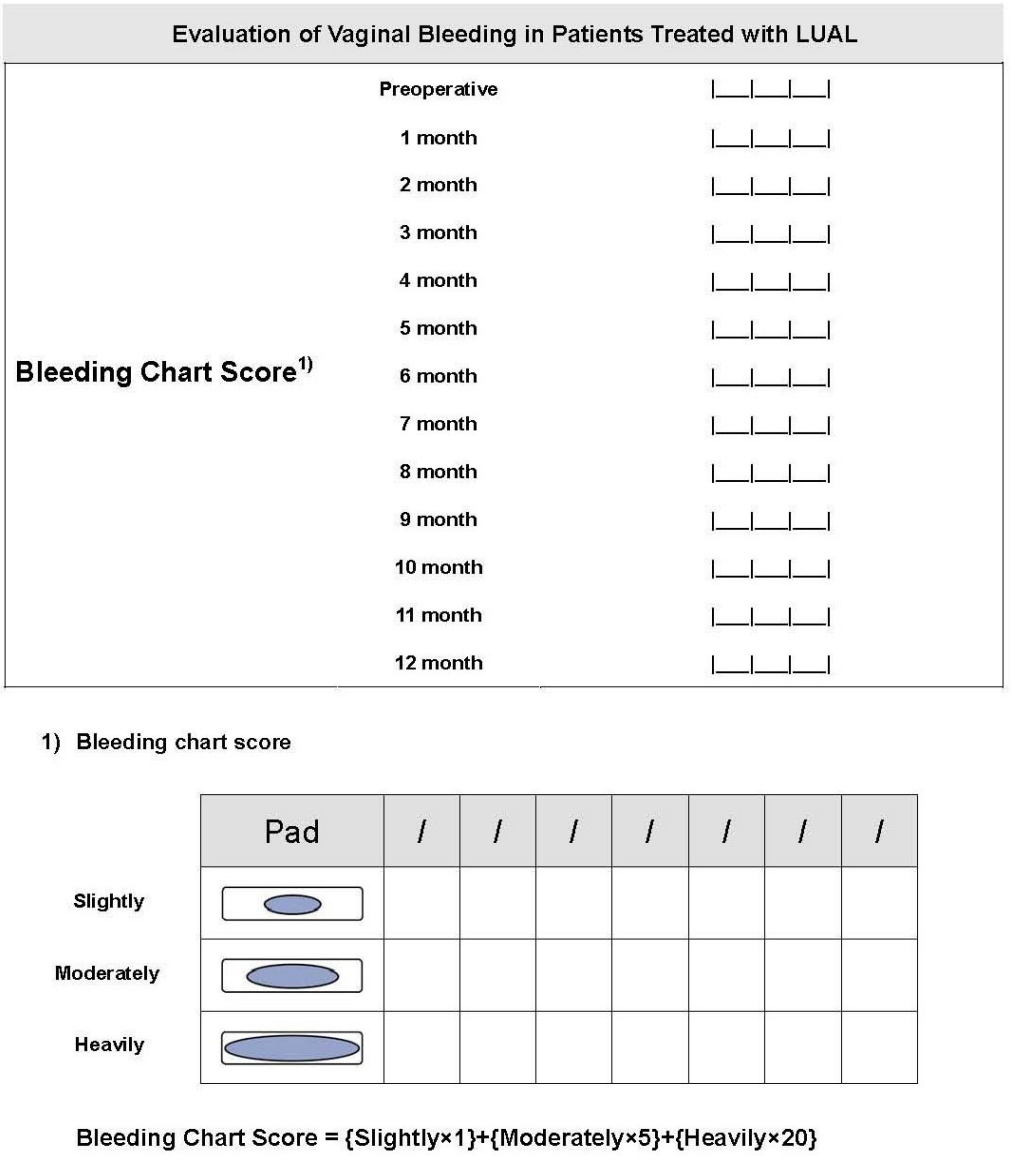
**Bleeding chart score by a simple visual assessment technique for evaluating vaginal bleeding in patients treated with laparoscopic uterine artery ligation**.

We have made "Bleeding Chart Score" by use of a simple visual assessment technique (Fig. 4) [10]. After we educate patients treated with LUAL, in particular, patients with preoperative symptoms such as menorrhagia and intermittent vaginal bleeding, about a simple visual assessment technique, they should record the amount of vaginal bleeding using the technique by her monthly. Thereafter, we calculate and record the amount of vaginal bleeding in the Bleeding Chart Score. After the completion of the current study, we will analyze the change of the amount of vaginal bleeding between preoperatively and 12 months after surgery.

#### Postoperative clinical outcomes

1) Anemia, hormonal status and serum CA-125 levels

Serum hemoglobin levels are evaluated in acute and chronic phases. In the acute phase, we check serum hemoglobin levels preoperatively and 2 days after surgery for comparing the effect of intraoperative blood loss between LUAL and LAVH. In the chronic phase, we check serum hemoglobin levels every 6 months, and we will compare serum hemoglobin levels between the 2 treatment methods.

The evaluation of hormone status is important because Liu et al [5] have reported that 3.5% of patients presented with elevated FSH, suggesting that LUAL can affect preoperative hormonal status. Moreover, serum CA-125 levels are increased in uterine fibroids, whereas they are decreased after the treatment of uterine fibroids. It means that serum CA-125 levels may be one of indirect markers reflecting the change of uterine fibroids [14]. Thus, serum LH, FSH, E2 and CA-125 levels are checked preoperatively and every 6 months, and we will compare these values between the 2 treatment methods.

2) Operation time, hospitalization and recovery time to routine life

The operation time is recorded in all patients for the investigation of whether there is a significant difference in the time between LAUL and LAVH. All patients are discharged as soon as possible if they are considered not to be hospitalized, and we will evaluate hospitalization and recovery time to routine life in all patients because the 2 parameters may reflect the time from surgery to healthy status.

3) Postoperative pain

Pain intensity is measured with a visual analogue scale (VAS), a 100-mm horizontal line with anchors of no pain and worst possible pain. The VAS is scored by measuring in millimeters the distance from the side marked no pain to the edge of the mark made by the patient. Possible scores ranges from a minimum of 0 to a maximum of 100 mm. The VAS scale is presented to the patients individually by instructed nurses who are unaware of assignments. After the VAS scales are evaluated during postoperative 3 days, the sums of scores will be compared between LUAL and LAVH.

4) Conversion to laparotomy

Although the 2 surgical treatments are similar in terms of laparoscopic managements, LUAL can preserve uterus with exploration of retroperitoneal space and ligation of uterine artery. On the other hand, LAVH removes uterus without the exploration of retroperitoneal space. Thus, these differences can contribute the rate of conversion from laparoscopic approach to laparotomy. We will compare the rate of conversion to laparotomy between LUAL and LAVH.

6) Satisfaction of postoperative sexual intercourse

Since patients treated with LUAL have cervices while those treated with LAVH have no cervices postoperatively, types of surgical treatment can affect postoperative sexual intercourse. Thus, the change of satisfaction of sexual intercourse will be evaluated after 12 months after surgery. The satisfaction of sexual intercourse is scored on 3-point scales, consisting of -1 ('not satisfied'), 0 ('no change') and +1 ('satisfied').

7) Complications associated with surgery

We divided complications associated with surgery as follows: intraoperative complication; complication during hospitalization; complication within 1 month; complication within 12 months. Moreover, the severity of the complications is defined as follows [9].

(1) Grade 1: no consequence for the patients and necessitated no or only nominal treatment (e.g. arterial spasm, postpuncture hematoma, surgical wound hematoma, vaginal discharge, urinary retension, thigh paresthesia).

(2) Grade 2: necessitated non-life-threatening additional treatment and had no sequelae of patients (e.g. urinary infection, severe pelvic pain, renoureteral colic, vulvovaginitis, anal fissure).

(3) Grade 3: death or complication that represents a threat to the patient's life or a source of permanent sequelae (e.g. death, deep vein thrombosis, transfusion, intraabdominal abscess, vesical fissure, surgical wound abscess).

After the completion of the current study, we will compare these results between the 2 treatment methods.

### Statistical analysis

We will analyze all patients assigned randomly. After informed consent is obtained, blocked randomization is performed by the Medical Research Collaborating Center (MRCC), a central office, in Seoul National University Hospital. The number of participants to be assigned to each of the comparison groups is balanced within blocks. The central office is remote from patient recruitment centers. Thus, participants' details are provided by phone, and the allocation sequence is concealed to individual staffing in the central office until a participant is irreversibly registered.

An intention-to-treat analysis will be performed as the least biased way to estimate intervention effects in the current study. For the primary endpoint, patients with missing EORTC-QLQ-C30 outcomes will be excluded from analysis because they cannot be considered to show specified minimum outcomes of the intended intervention. Analysis of covariance (ANCOVA) will be applied to compare the QOL scores on the basis of EORTC QLQ-C30 between the 2 treatment methods, adjusting for baseline values. After we consider the overall baseline EORTC score, a level of p < 0.05 will be assumed that it indicates imbalance. Thereafter, we will declare the variable to be imbalanced, and then include baseline values of EORTC QLQ-C30 in the ANCOVA to adjust the final results.

Other comparisons between the 2 groups will be made with the use of a two-sided Student's t-test and the Mann-Whitney *U *test for continuous data and the chi-square test for categorical data. The original power calculation requires the enrolled of a total 200 patients to give a power of 90% to detect a difference of 10% in the EORTC QLQ-C30 score at 12 moths (the primary outcome) at the 0.05 significance level.

All independent data such as serious adverse events and recruitment rates will be reviewed by MRCC and the Institutional Review Boards (IRBs) in Seoul National University Hospital, Seoul National University Borame Hospital and Seoul National University Bundang Hospital every 6 months. Moreover, the informal interim analysis for preliminary results will be undertaken, which will inform us whether the current study will be continued or not and maintain the quality of data through balancing baseline data.

### Publication policy

The result of the current study will be submitted for publication to peer-review medical journals regardless of whether the outcome is in favor of the objectives in the current study.

### Approval by the Institutional Review Board

We obtained the approval of the IRB of Seoul National University College of Medicine/Seoul National University Hospital for the current study in advance, and the IRB number is H-0704-032-205.

### Registration of clinical trial

The current study has been registered in the Current Controlled Trials with the title "A randomized study of the postoperative quality of life: Laparoscopic uterine artery clipping versus laparoscopy-assisted vaginal hysterectomy for the management of symptomatic uterine fibroids (UTAC trial)" (Trial No. ISRCTN 6790866) [15].

### Trial timetable

#### Trial start

July, 2007

#### Trial recruitment completed

June, 2008

#### Trial end

June, 2009

#### Trial duration

2 years

#### Duration of each patient's participation

1 year

## Discussion

The current study has been designed to compare postoperative QOL between LAUL and LAVH, and to show the similar efficacy of LAUL, compared with LAVH, for the treatment of symptomatic uterine fibroids. Except for LUAL, there are other surgical methods for the treatment of symptomatic uterine fibroids as follows.

Firstly, myomectomy can be performed for reproductive women with uterine fibroids as a cause of infertility and patients with symptomatic uterine fibroids who want to preserve uterus. Although many clinicians have suggested that myomectomy might have more chance of intraoperative bleeding and postoperative transfusion than hysterectomy, some studies have shown that there is no difference of bleeding and transfusion between myomectomy and hysterectomy [16,17]. However, myomectomy can lead to pelvic adhesion which may inhibits conception, and the rate of recurrence is up to 50%. Thus, one third of all patients are known to undergo re-operation for the treatment of uterine fibroids [18].

Secondly, myolysis had been introduced in 1993 as an alternative method for treating symptomatic uterine fibroids. Moreover, it can be performed by laparoscopy or transvaginal approach using thermomyolysis by Nd:YAG laser, cryomyolysis and myoma interstitial thermo-therapy. Although the size of uterine fibroids has been reported to decrease by 30–50%, severe pain by necrosis of them, uterine rupture during pregnancy, uterine abscess and pelvic adhesion can be developed [19,20].

Thirdly, uterine artery embolization (UAE) has also been introduced in 1995 for treating symptomatic uterine fibroids [21]. Early analysis of an open, prospective, voluntary U.S. registry including 3,160 of patients revealed major complications in 5.5% of patients at 30 days, with 0.1% requiring a hysterectomy [22]. Moreover, the National Institute for Health and Clinical Excellence issued guidelines in October 2004, starting that the procedure appeared to be safe for routine use and that the majority of patients have short-term symptomatic relief [23]. However, the technique has some limitations such as postembolization syndrome, the time required to perform the procedure, and radiation exposure for preventing the widespread application.

Thus, LUAL can be efficient for the treatment of symptomatic uterine fibroids, overcoming the complications of UAE. Nevertheless, there is a need for careful assessment of the effects of the procedure on QOL, particularly in comparison with hysterectomy, especially, LAVH.

In conclusion, the current study is meaningful in that we can suggest the results of the comparison of postoperative QOL between LUAL and LAVH. If there is no difference of postoperative QOL between the 2 treatment methods for the treatment of symptomatic uterine fibroids, the comparison of QOL between LUAL and UAE will be also needed as a surgical treatment for preserving uterus.

## Abbreviations

LUAL: laparoscopic uterine artery ligation; LAVH: laparoscopy-assisted vaginal hysterectomy; QOL: quality of life; GnRH: gonadotropin-releasing hormone; EORTC QLQ-C30: European Organization for Research and Treatment of Cancer Quality of Life Questionnaire for Cancer Patients version 3.0; USG: ultrasonography; PID: pelvic inflammatory disease; VAS: visual analogue scale; MRCC: Medical Research Collaborating Center; IRB: Institutional Review Board; UAE: uterine artery embolization.

## Competing interests

The authors declare they have no competing interests.

## Authors' contributions

HSK designed this randomized controlled trial, and performed preliminary data analysis, and HSK and MKK and HHC wrote this manuscript. JWK is the chief investigator, and designed this randomized controlled trial, and contributed to writing this manuscript, and managed patients according to UTAC trial protocol. TSL and YTJ and YBK cooperated in designing this randomized controlled trial, and managed patients according to UTAC trial protocol. HWJ and NHP and YSS and SBK commented upon the design of this randomized controlled trial, and managed patients according to UTAC trial protocol.
